# Clinician and computer: a study on doctors’ perceptions of artificial intelligence in skeletal radiography

**DOI:** 10.1186/s12909-022-03976-6

**Published:** 2023-01-10

**Authors:** Thomas James York, Siddarth Raj, Thomas Ashdown, Gareth Jones

**Affiliations:** 1 Alexander Fleming Building, Imperial College London, South Kensington Campus, London, UK; 2 King’s College London, London, UK; 3 Guy’s and St Thomas’ Hospitals NHS Trust, London, UK; 4 Imperial College Healthcare NHS Trust, London, UK

**Keywords:** Artificial intelligence, Skeletal radiology, Computing methodologies, Decision support systems, clinical

## Abstract

**Background:**

Traumatic musculoskeletal injuries are a common presentation to emergency care, the first-line investigation often being plain radiography. The interpretation of this imaging frequently falls to less experienced clinicians despite well-established challenges in reporting. This study presents novel data of clinicians’ confidence in interpreting trauma radiographs, their perception of AI in healthcare, and their support for the development of systems applied to skeletal radiography.

**Methods:**

A novel questionnaire was distributed through a network of collaborators to clinicians across the Southeast of England. Over a three-month period, responses were compiled into a database before undergoing statistical review.

**Results:**

The responses of 297 participants were included. The mean self-assessed knowledge of AI in healthcare was 3.68 out of ten, with significantly higher knowledge reported by the most senior doctors (Specialty Trainee/Specialty Registrar or above = 4.88). 13.8% of participants reported an awareness of AI in their clinical practice.

Overall, participants indicated substantial favourability towards AI in healthcare (7.87) and in AI applied to skeletal radiography (7.75). There was a preference for a hypothetical system indicating positive findings rather than ruling as negative (7.26 vs 6.20).

**Conclusions:**

This study identifies clear support, amongst a cross section of student and qualified doctors, for both the general use of AI technology in healthcare and in its application to skeletal radiography for trauma. The development of systems to address this demand appear well founded and popular. The engagement of a small but reticent minority should be sought, along with improving the wider education of doctors on AI.

## What is already known on this topic

Published research has indicated favorable patient attitudes for the development of AI technology to assist their care providers with interpreting MSK radiographs for trauma. However, there has so far been very limited study on the views of clinicians towards this potential solution to an increasing burden of trauma imaging.

## What this study adds

This study presents novel data on the opinions of a wide cross section of interpreting clinicians. Although existing knowledge of the area was limited, there was evidence of strong support amongst training and qualified doctors for AI technology that examines MSK trauma X-rays. This was found for both identification of positive findings and confirmation of negative imaging.

### How this study might affect research, practice, or policy

These findings serve to validate continued research and development in this topical clinical field, with clear stakeholder support now established in both the end-users and beneficiaries of this hypothetical technology.

## Background

### The Burden of Skeletal Radiology

Musculoskeletal (MSK) conditions account for over 22% of morbidity in the UK [1]. They are responsible for 25% of surgical interventions [2], up to 60% of emergency presentations, and 7.9% of hospital admissions [1, 3]. As the first-line investigation for many such injuries, 23.2 million plain radiographs are ordered per annum, representing 58.6% of all imaging tests in England [4]. Only 28% of those requested in accident & emergency (A&E) are reported the same day, with just 53% reported the next, showing the challenge of coping with high volumes of radiographs [4].

Interpreted by either doctors or specialist nursing staff, studies have shown significant disparity in assessment due to varying training and seniority amongst assessors. Factors such as physician-fatigue and workload have also been tied to an increased risk of misdiagnosis, a recent census indicated short-staffing amongst radiologists by as much as 33% [5].

Whilst the Royal College of Radiologists has stressed the importance of improved recruitment and retention, modelling suggests that these measures will fail to meet the increased demand. The Consultant short fall is expected to grow to 44% by 2025 [5]. These issues have been compounded by pressures exerted on the UK’s health system by COVID-19; 41% of Radiologists reported being moderately or severely demoralised [5, 6].

### Imperfect solutions

To address these pressures, Teleradiology services have been increasingly used in urgent and out of hours reporting. Radiological images are sent away for interpretation, often overseas, with intended savings due to time difference and equivalence of training. However, concerns over patient safeguards and the impact on local radiology services have been identified by professional bodies in the UK, Europe, and North America [7].

Virtual Fracture Clinics (VFCs) also allow for remote non-urgent imaging review, acting to confirm or refute an initial emergency department assessment. Typically involving a consultant orthopaedic surgeon and MSK specialist radiologist [8], these have not been found to enhance overall reporting capacity but have become increasingly relied upon to reduce burden on in-person orthopaedic fracture clinics [9].

### The role of artificial intelligence

Increasingly, research has looked for novel solutions to satisfy this demand for reporting provision. One area of focus has been Artificial Intelligence (AI). Computer assistance in radiology dates to 1963, when data processing was used to identify the increased density of lung cancers on chest X-rays [10]. This was a rudimentary system, not diagnostic, but its speed and accuracy demonstrated the usefulness of AI in radiology. More recently, AI has been applied to the identification of tuberculosis interstitial lung disease [11]. In pneumonia detection, it has achieved a higher rate of accuracy than radiology registrars [12]. Limited work has already been conducted to examine the complex relationship between these systems and the clinicians who use them [13].

Specific to MSK imaging, convolutional neural networks have been successfully used to detect vertebral fractures at a noninferior rate to orthopaedic surgeons [14]. There has been a limited but growing use of the technology in the appendicular skeleton [15], with an application towards arthroplasty starting to become more prevalent [16].

Parallel to academic development, the market in medical imaging AI has grown substantially in recent years, with a forecast compound annual growth rate of 30.4% from 2021 to 2026. By 2025 the global market is expected to exceed $181.1 million [17]. The NHS has ring fenced £140 million for the development of AI in response to operational challenges, and it is cited as a modernising tool with potential for cost saving in the NHS long term plan [18].

Despite this potential, several factors hinder the application of AI in radiology. The integration of AI systems with legacy PACS software has proved challenging [19], as has assigning medico-legal liability to decision-making AI tools [20].

### Aims and objectives

With growing utilization of AI in skeletal radiology, this study sought to establish novel data on the perceptions of qualified and student doctors (medical students) towards the technology. Specifically, by investigating their pre-existing knowledge of AI (in and out of hospital), their confidence in interpreting plain radiographs for skeletal trauma, and their projected confidence in using a system to identify positive and negative X-rays. Further consideration was given to how demographic features, principally the participant’s training grade, might affect these perceptions.

## Methods

### Ethics and approval

The design for this study underwent prospective approval by Guy’s and St Thomas’ National Health Service (NHS) Trust. No Research Ethics Committee review was required as no patient or volunteer intervention was performed. All data was handled in accordance with General Data Protection Regulations (GDPR) [21] and the study was conducted as required by the National Institute for Health Research’s Good Clinical Practice (GCP) guidelines for clinical research [22].

### Questionnaire design

Following a scoping review of the existing literature, a novel questionnaire was designed using validated survey methodology [23, 24]. The questionnaire can be found in full as *Appendix 1.* Although all respondents were medical professionals, plain English was used where possible to minimise ambiguity and misinterpretation; guidelines from the National Council for Voluntary Organisations were used as a benchmark for this [25].

The first three questions identified foundational, demographic information on study respondents. These allowed assessment of the representativeness to the broader population of student and qualified doctors.

The following two questions (each divided into two components) established participants’ self-assessed comprehension of AI technology, along with their exposure to it in a healthcare setting. A Likert-Scale [24], where ten represented ‘extremely knowledgeable’ and one represented ‘not knowledgeable at all’ was used for the first pair of these.

The sixth question was divided into three components and sought to identify the participants confidence in interpreting plain skeletal radiographs for trauma, examining commonly cited factors which negatively influence the process of initial interpretation.

The seventh question identified key capabilities required to perform the initial interpretation of plain skeletal radiographs using Picture Archiving Communications Systems (PACS), and asked respondents to indicate their confidence in this.

The remaining questions were used to assess the favorability of respondents towards the general application of AI to healthcare, and to its specific use-case in trauma radiography. This was further assessed regarding capacity to indicate positive/negative findings.

These 11 questions were compiled into a *Google Form*, allowing for distribution and for participants to enter responses remotely. The *Google Form* handled data in accordance with industry standard security protocols [26] and took no ownership over the collected data [27].

Formal validation of the questionnaire was not possible due to the time constraints imposed on the data-collection period. However, usability testing was performed on a panel of sample participants to assess potential issues. The average time for completion was six minutes and 20 s. The authors reviewed the answers from the sample participants in order to assess face and content validity, the scope of the questionnaire was deemed sufficient to capture the required data. No issues with question phrasing or completion were identified.

### Questionnaire distribution

The questionnaire was distributed to student and qualified doctors across Greater London and the Southeast of England, using hospital and university networks to disseminate the Google Form.

Validation of participants was performed by requiring an NHS or medical school email address prior to completion of the questionnaire. Participants were also asked to confirm that they were currently involved in the interpretation of MSK trauma radiology, or actively being trained in the practice. Participants could elect to register for the chance to receive a nominal prize on completion of the questionnaire (a single £50 gift voucher). It was made clear that this would be randomly allocated, and the responses given had no bearing on the recipient of the prize.

### Database generation and statistics

Questionnaire results were exported from *Google Forms* into the *IBM SPSS 28 Academic Edition* software package. This was used to perform data handling and statistical interrogation of the results, T-test and Pearson’s Correlation Coefficient were used as the principal reporting measures. A conventional value of *p* < 0.05 was taken to indicate statistical significance. *Google Enterprise Cloud Natural Language API* was used to perform natural language analysis and identify key themes from the free text responses in questions *5.b* and *6.c see Appendix 1*.

## Results

### Demographics (Questions One to Three)

During the period from 5^th^ January 2021 to 5^th^ August 2021, the Google Form was accessed by a total of 353 unique users. From these, 54 respondents did not meet the validation requirements described in *Questionnaire Distribution*, by failing to confirm their adequate involvement in MSK trauma radiology. This left 299 eligible participants who completed the questionnaire. 297 did so in a manner which enabled data interpretation. The responses of these participants were included in their entirety, no additional source of primary data was employed.

Of the participants, 151 identified themselves as male (50.8%), 144 were female (48.5%), one non-binary/other (0.3%), and one preferred not to say (0.3%). The youngest male participant was 19 years old, the oldest 64 (mean 28.50). Female participants ranged from 18 to 42 years old, being on average slightly younger than their male counterparts (mean 25.61) see Table 1.

**Table 1:** Gender & Age

*Gender*	*Number of Respondents*	*Mean Age*
*Male*	151	28.50
*Female*	144	25.61
*Non-binary/other*	1	31
*Prefer not to say*	1	30

The training group most represented amongst participants was Foundation Year One doctors, 108 (36.4%) of the total. The group least represented amongst participants was Foundation Year Two doctors, 28 (9.4%) of the total see Table 2.

**Table 2:** Level of Training

*Training Grade*	*Number of respondents*	*Percentage of Respondents*
*Medical Student*	77	25.93%
*FY1*	108	36.36%
*FY2*	28	9.43%
*ST/CT 1–2*	35	11.78%
*ST3/SpR or Above*	49	16.50%

### Familiarity with Artificial Intelligence (Question Four)

When asked to indicate their degree of knowledge of AI technology in general, the overall participant mean was found to be 4.12 see Table 3. The lowest mean knowledge was identified in Foundation Year One doctors, scoring a mean of 3.63. This was significantly lower than the population mean, *t*(107) = -2.01, *p* = 0.023.

**Table 3:** Questionnaire Mean Responses (Confidence Intervals)

Tr*aining Grade*	*Self-Assessed Knowledge of AI—General*	*Self-Assessed Knowledge of AI—Healthcare*	*Confidence in Reporting*	*Confidence with PACS*	*Agreement-General Benefit*	*Agreement—MSK Trauma Radiographs*	*Confidence – Positive Findings*	*Confidence – Negative Finding*
*Medical Student*	4.1 (3.385 – 4.815)	3.56 (2.845 – 4.275)	4.9 (4.185–5.615)	3.78 (3.065 – 4.495)	8.05 (7.335 – 8.765)	7.97 (7.255 – 8.685)	7.52 (6.805 – 8.235)	6 (5.285 – 6.715)
*FY1*	3.63 (3.026 – 4.234)	3.09 (2.486 – 3.694)	5.23 (4.626–5.834)	5.96 (5.356 – 6.564)	7.9 (7.296 – 8.504)	7.94 (7.336 – 8.544)	7.19 (6.586 – 7.794)	6.12 (5.516 – 6.724)
*FY2*	4.46 (3.275 – 5.645)	4.04 (2.855 – 5.225)	6.57 (5.385–7.755)	7.29 (6.105 – 8.475)	7.58 (6.395 – 8.765)	7.21 (6.025 – 8.395)	7.21 (6.025 – 8.395)	6.43 (5.245 – 7.615)
*ST/CT 1–2*	4.26 (3.200 – 5.320)	3.83 (2.770 – 4.890)	6.29 (5.230–7.350)	6.89 (5.830 – 7.950)	7.83 (6.770 – 8.890)	7.69 (6.630 – 8.750)	7.34 (6.280 – 8.400)	6.89 (5.830 – 7.950)
*ST3/SpR or Above*	4.94 (4.044 – 5.836)	4.88 (3.984 – 5.776)	7.49 (6.594–8.386)	7.67 (6.774 – 8.566)	7.71 (6.814 – 8.606)	7.33 (6.434 – 8.226)	6.94 (6.044 – 7.836)	6.08 (5.184 – 6.976)
*Overall*	4.12 (3.756 – 4.484)	3.68 (3.316 – 4.044)	5.77 (5.406–6.134)	5.91 (5.546 – 6.274)	7.87 (7.506 – 8.234)	7.75 (7.386 – 8.114)	7.26 (6.896 – 7.624)	6.2 (5.836 – 6.564)

The most senior training group, ST3/SpR or Above, were also those who reported their knowledge of AI technology to be the highest, mean of 4.94. This was found to be significantly higher than the overall mean, *t*(48) = 2.47, *p* = 0.014.

The mean, overall participant self-assessment for their knowledge of AI technology, applied to the healthcare setting was 3.68 see Table 3. This was found to be significantly lower than for AI technology in general, *t*(296) = -2.50, *p* = 0.013.

FY1 doctors reported the lowest level of knowledge (mean 3.09), significantly below that of the overall mean, *t*(107) = -2.53, *p* = 0.012. Again, doctors in the ST3/SpR or Above category reported the highest knowledge of AI in healthcare, (mean 4.88), significantly above that of participants overall, *t*(49) = 3.50, *p* =  < 0.001.

### Use of AI Technology in Clinical Practice and Training (Question Five)

**Fig. 1:**
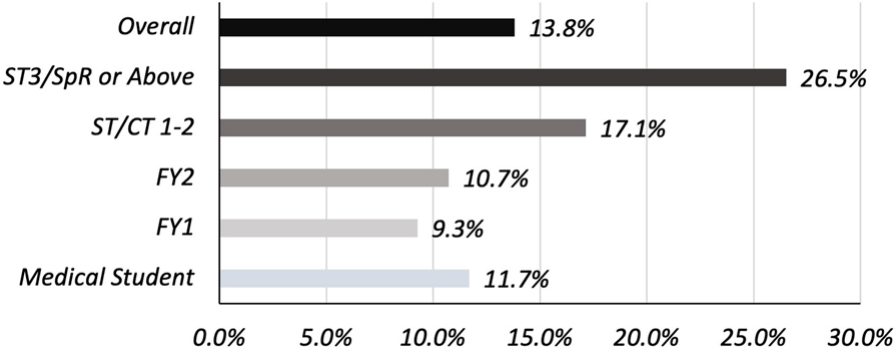
Percentage Aware of AI Technology in Their Practice

Of the total 297 participants, 41 (13.8%) indicated that they were aware of the use of AI technology in their current or recent clinical work. Amongst FY1 doctors, 10 (9.3%) indicated such an awareness. The group reporting the most exposure to healthcare AI in their work was ST3/SpR or Above doctors, with 13 (26.5%) saying they were aware of such technology, see Fig 1.

Natural-language, text analytics software was applied to the 41 free text responses to question 5.b, which asked participants indicating an awareness of AI technology in their current or recent clinical practice to elaborate.

Cardiac electrophysiology was the most identified area of AI application (eight participants, 19.5%), specific mention of electrocardiogram interpretation was present in all of these. Diagnostic radiography was mentioned by seven participants (17.1%); mammography, chest radiographs, vertebral fractures, and computed tomography for trauma were all identified. The use of AI technology in pre-operative radiography was noted by five participants (12.2%). Of these mentions, orthopaedic surgery (specifically templating for hip arthroplasty) was noted by three participants. Other mentions were for urological and neurosurgical procedures. Three participants (7.3%) noted the use of AI in identifying biomarkers in oncology, and two (4.9%) its use to alert clinicians to acute kidney injury from biochemistry lab results. Nine respondents (22.0%) made reference to AI technology but without sufficient explanation for data classification e.g. ‘machine learning’.

### Interpreting Plain Film Radiographs for Skeletal Trauma (Question Six)

The mean confidence in ability to report plain skeletal radiographs for trauma was 5.77, se Table 3. Amongst medical students this fell to 4.90, significantly lower than the overall average *t*(76) = -3.63, *p* < 0.001.

ST3/SpR or Above doctors were found to report the greatest confidence in their ability to perform this task; mean of 7.49, significantly higher than the mean across all respondents *t*(49) = 5.90, *p* < 0.001.

There was found to be a statistically insignificant, weakly positive correlation between increased seniority in training grade, and confidence in reporting *r*(295) = 0.055, *p* = 0.35.

The factor most identified as the principal influence on the ability to accurately assess these radiographs was time-constraint, accounting for 37.0% of participant responses see Table 4. Fatigue was identified by 22.6% of participants, closely followed by the 20.5% of people who felt their ability was unaffected by any such factors. Of those 22 participants reporting ‘other’ (7.4%), a lack of training or exposure to the imaging was identified in all but a single case.

**Table 4:** Principle External Factor Influencing Interpretation

*Influencing Factor*	*Number Affected*	*Percentage Affected*
*Time-constraint*	110	37.0%
*Fatigue*	67	22.6%
*Stress*	37	12.5%
*Other*	22	7.4%
*I don't feel that my ability is affected by these factors*	61	20.5%

### Confidence Using PACS Software (Question Seven)

Participants reported a mean confidence in using PACS software of 5.91 see Table 3. Mean confidence was significantly lower amongst medical students, 3.78; *t*(76) = -6.38, *p* < 0.001. Again, ST3/SpR or Above doctors reported the greatest confidence in using their hospital’s PACs software (mean 7.67), this was significantly higher than the overall average *t*(48) = 4.45, *p* < 0.001.

A significant, weakly positive correlation between increasing seniority and confidence in using PACS software was observed, *r*(295) = 0.484, *p* < 0.001.

### The General Role of AI Technology in Healthcare (Question Eight)

**Fig. 2:**
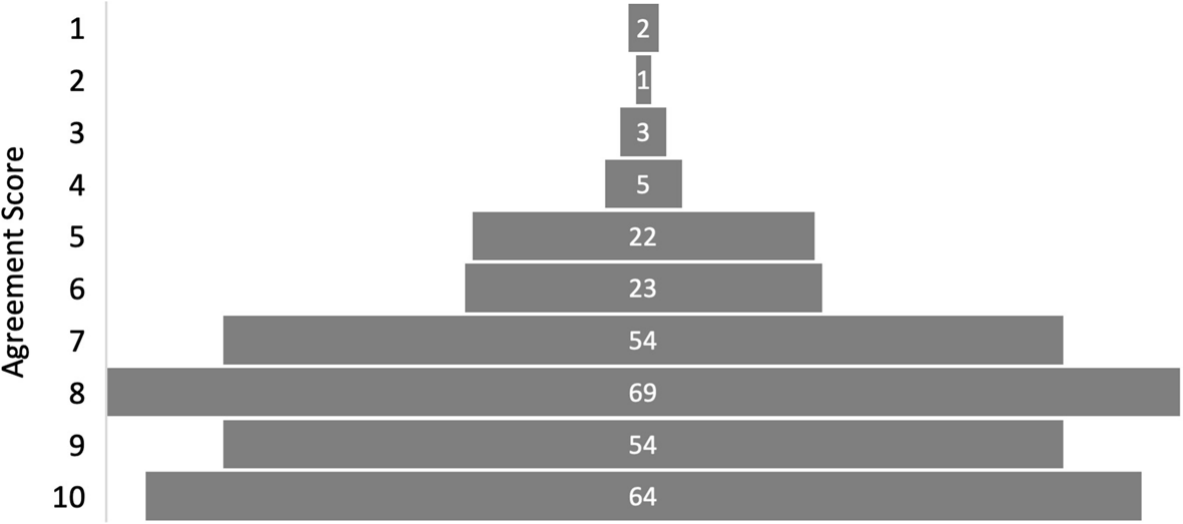
Participant Responses – AI Technology will be of General Benefit to Healthcare

In response to being asked to what extent they agreed that, in general, AI would be of benefit to healthcare, the overall mean score was 7.87 ± 0.364 (± 4.6%) [7.506 – 8.234] see Table 3, with a modal response of eight, see Fig. 2. Although variations were seen in the average of different training grades, with FY2 doctors scoring the lowest and Medical Students scoring the highest, these were not found to be statistically significant. No correlation was observed between seniority and agreement with the question posed.

### AI Technology in Plain Skeletal Radiographs for Trauma (Question Nine)

**Fig. 3:**
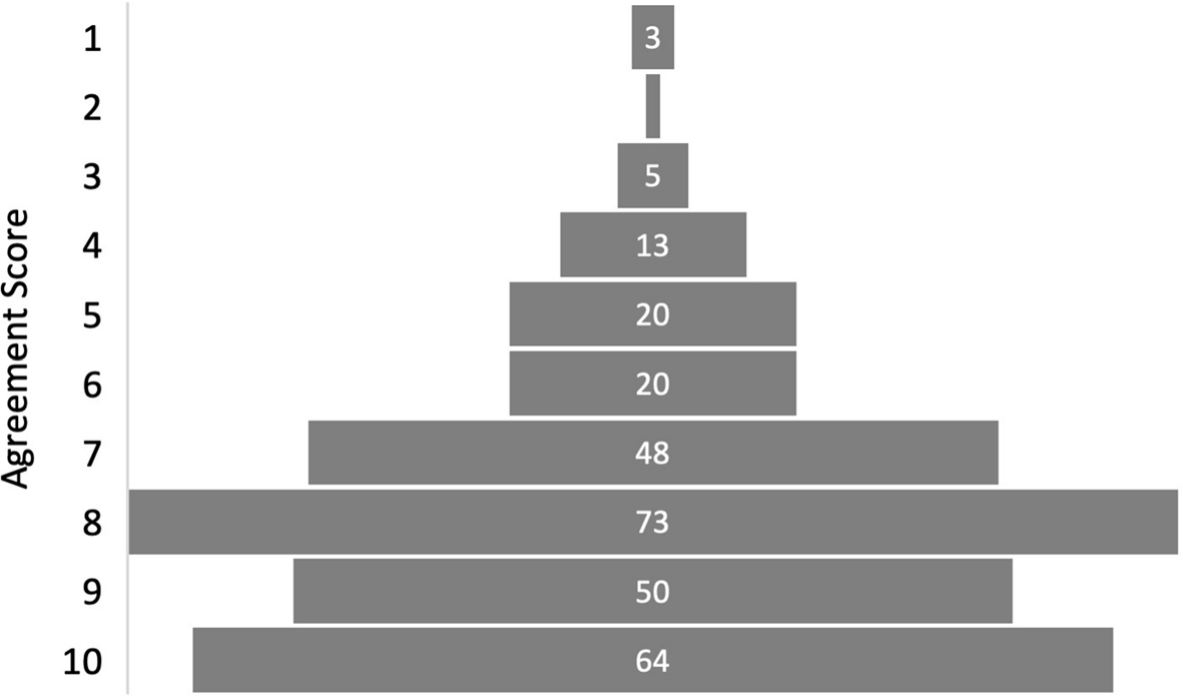
Participant Responses – AI system for interpreting trauma MSK radiographs

The mean reported value for the extent of agreement that an AI system would assist with interpreting trauma MSK radiographs was 7.75, see Table 3. The modal response to question nine was a score of eight, see Fig. 3. No significant variation was seen between training grades and no significant correlation was found between level of seniority and agreement with the question.

### Identifying Positive Findings (Question Ten)

**Fig. 4:**
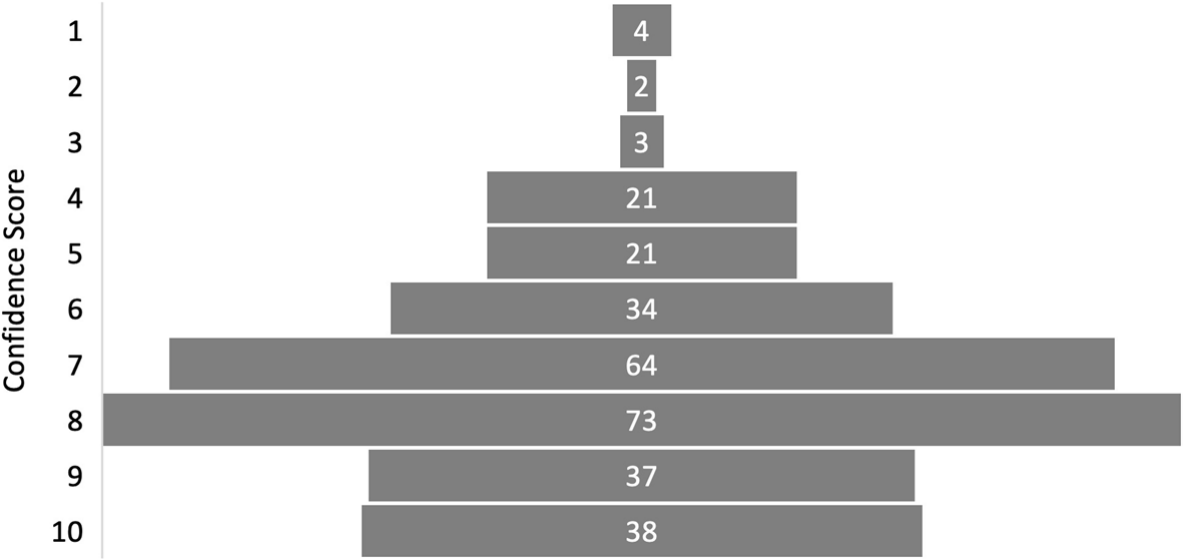
Participant Responses – Confidence for Positive Findings

When asked to identify their confidence in using an AI system to identify and label potential positive findings, the mean response was 7.26, see Table 3. The modal response to this question was again a confidence score of eight, recorded by 73 participants (24.6%), *see *Fig. 4.

ST3/SpR or Above doctors had the lowest mean response (6.94), and Medical Students had the highest (7.52). Despite this, there was not found to be statistically significant variation from the overall average in any individual training grade’s responses. There was however a weakly negative correlation between increased training grade and confidence in the concept, *r*(295) = -0.12, *p* = 0.036.

### Identifying Negative Imaging (Question 11)

**Fig. 5:**
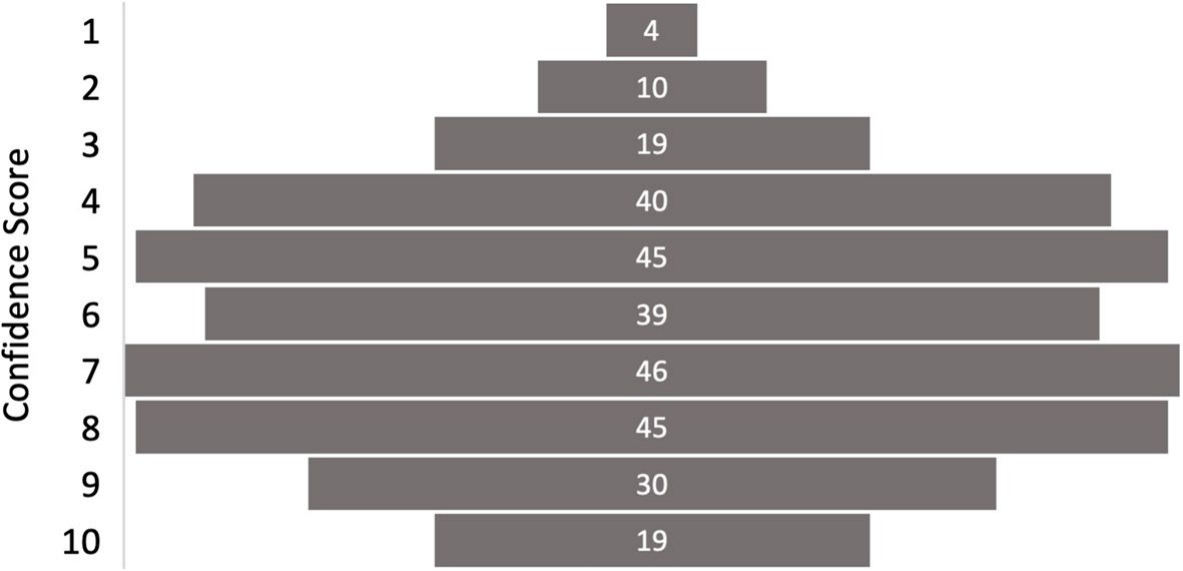
Participant Responses – Confidence in Supporting Identification of Negative imaging

The overall, mean confidence for a system which identified imaging as negative for significant findings was 6.20, see Table 3. The modal confidence score for this question was seven, recorded by a total of 46 participants (15.5%), see Fig. 5.

Medical Students reported the lowest confidence (mean 6.00), and ST/CT 1–2 doctors reported the highest confidence (mean 6.89). There was, however, no statistically significant variation between training grades and nor was there a correlation between seniority and confidence in a system to identify negative imaging.

## Discussion

### Demographic Features

The 297 participants whose data is reported on in this study are considered demographically representative of the broader physician work force in the United Kingdom (UK). The approximately equal gender divide is concordant with GMC records of the 300,687 registered doctors, of whom 160,178 (53.3%) were male and 140,509 (46.7%) were female [28].

### Familiarity with artificial intelligence

Questionnaire participants reported a mean knowledge of AI technology in general (excluding applications in healthcare) of 4.12 out of a possible score of 10. This indicates a general lack of knowledge amongst doctors, a finding supported by previous research which has shown as few as 50% are aware of either the term machine learning or deep learning [29]. Such a finding would not be out of step with the general population, where an in-depth knowledge of AI is far from common place. Polling in the UK has found that only one tenth of the population reported knowing a lot about AI [30].

The most senior training grade (ST3/SpR or Above) reported the highest average score, with a mean of 4.94. This seems to contradict the frequently reported theory that younger adults are more knowledgeable of novel technology due to their parallel development with it [31]. The frequency with which medical professionals interact with new technologies may explain this observation.

When participants indicated a knowledge of AI in healthcare, a mean rating of 3.68 out of 10, was observed. This indicates a lower clinical knowledge of AI than general awareness of the technology. A lack of clinician understanding has previously been identified as a significant barrier to the adoption of AI systems in healthcare. One prominent example of this has been IBM’s *Watson for Oncology* which is able to offer expert treatment advice for 12 of the most common malignancies [32]. Despite promising trial data[33], clinicians have struggled to adopt the software into their practice—being unclear of its utility when concordant with their clinical assessment but reticent to accept a differing opinion, the rationale for which was not apparent to them [34].

Increasing exposure to AI in medical curricula has been identified as an important means of bridging this knowledge gap [35]. The finding that FY1 doctors (the most junior grade of qualified doctor in the UK healthcare system) reported the lowest mean knowledge of AI in healthcare, suggests that this deficiency is not yet being met in their medical school training.

### Exposure to AI Technology in Clinical Practice and Training

Overall, 13.8% of participants indicated that they were aware of using AI in their clinical practice. This is indicative of sparce uptake of the technology in the UK healthcare system. Despite substantial governmental focus and funding through NHSx [18], independent reports have revealed delayed adoption of the technology in the health service [36, 37]. One cause for this may be reticence from trusts to expose themselves to a highly complex regulatory environment. The implementation of a health service-wide strategy for this, such as that now set out in The Topol Review [38], is considered to be an important step towards building a more digitally-competent clinical workforce.

As with many jurisdictions, the UK is yet to develop a statutory definition of AI [39] and Trusts have already fallen foul of regulators due to their involvement with the technology [40]. A clear legal framework is essential to encourage the wider uptake of the technology; much of it having so far been developed in a largely unregulated landscape.

A significant degree of variability was seen in the exposure of different training grades to AI, with greater than one in four ST3/SpR or Above doctors reporting that they were aware of using it in their practice. This finding likely supports the earlier hypothesis that greater exposure to AI may have resulted in this cohort self-reporting the highest level of knowledge for the technology, both in general and in a healthcare setting.

### Optimism for AI in Healthcare

Participants indicated a clear belief that AI would broadly be of benefit to healthcare provision. Despite variation in exposure to the technology, such a finding has been observed by research conducted amongst doctors in other countries. One international study of artificial intelligence in psychiatry found a similar overall level of confidence in AI, but noted that physicians in the UK were more certain that the benefits of adopting such technology would outweigh the risks [41]. Research has also indicated that UK doctors may possess a more positive perception of AI technologies than their global peers [42].

A small minority of participants were reticent about AI technology in healthcare, with two stating that they felt it would be of no benefit whatsoever. Whilst it may be the case that the objections of these sceptical participants represent normal variation in technology adoption, a pro-innovation bias should be guarded against [43]. The concerns of these AI-sceptics should be closely examined in the development of such technologies.

### AI in MSK Trauma Radiographs

A high level of agreement was reported for the statement that an AI system would assist with interpreting trauma MSK radiographs. This suggests there is clear support amongst relevant student and practicing clinicians for the development of AI technologies which address this need. This finding was consistent across the entire assessed spectrum of seniority, with no significant variation between training grades, suggesting that both current and future reviewers of trauma MSK radiographs see value in using AI to assist their interpretation.

The support for AI in MSK trauma patients has also been found to be substantial [44], stakeholder backing is therefore identified amongst both beneficiaries, and users of the technology. To complete this assessment of favourability, further research amongst orthopaedic and radiology key opinion leaders is indicated.

### Limitations

There was noted to be substantial variation in the number of participants from each training grade. Whilst every effort was made to reach a representative sample of student and qualified doctors, there is a known propensity for this study’s distribution methodology to result in participants mirroring the demographics of the distributors [45]. Despite this, sufficient participation from each group was obtained to identify significant variation across training grades.

A further weakness of this study was the difficulty in clearly establishing what was meant by *AI technology applied to skeletal radiographs for trauma*. As the questionnaire was addressing a hypothetical software, it was not possible to fully describe capabilities or integration with existing systems. Whilst a degree of understanding was presumed for participants based on their professional experience, it is plausible that some did not clearly appreciate what was meant by AI technology. Although a relatively minimal level of technical literacy was required to answer this questionnaire, the low self-reported knowledge of participants for AI potentially suggests this as a limitation of the study design.

## Conclusions

A generally low knowledge of AI was reported by participants, with a significantly poorer self-assessed understanding of AI applied to healthcare than in its general application. This indicates a requirement for additional training in medical curricula; a crucial step if clinicians are to be able to scrutinise products which look certain to become an increased component of their practice.

Those representing the most senior training grade, reported the highest knowledge of AI technology, and a broad range of AI applications were identified in clinical practice; the most common being electro-cardiology but with awareness of uses in mammography, chest radiographs, and oncology.

In considering the role of AI technology in healthcare, the significant majority of both student and qualified doctors held a generally favourable view. With a mean score of 7.87 and modal score of eight out of ten, participants were very clear in their belief that AI technology would be of benefit to healthcare. Only a very limited number (just two participants) were in strong disagreement with this statement.

Specific to the development of AI in assisting trauma MSK radiograph interpretation, a significant degree of enthusiasm was noted, with high levels of agreement to the question statement seen across all training grades. Although substantial agreement was observed in both cases, slightly greater support was noted for a system which would identify positive findings rather than one aiming to classify an image as negative for significant findings.

These findings represent compelling evidence that a broad spectrum of clinicians support the development of AI in skeletal radiography for trauma. Whilst there is a substantial body of published research [15], there remains a limited provision of the technology for clinical practice. The long-established challenges of high volume and complex imaging, make a persuasive case for development in this area.

## Data Availability

The datasets used and/or analysed during the current study are available from the corresponding author (in anonymized form) on reasonable request.

## Abbreviations

MSK: Musculoskeletal
A&E: Accident & emergency
VFCs: Virtual Fracture Clinics
AI: Artificial Intelligence
NHS: National Health Service
GDPR: General Data Protection Regulations
GCP: Good Clinical Practice
PACS: Picture Archiving Communications Systems
UK: United Kingdom
